# Revealing Tumor Habitats from Texture Heterogeneity Analysis for Classification of Lung Cancer Malignancy and Aggressiveness

**DOI:** 10.1038/s41598-019-38831-0

**Published:** 2019-03-14

**Authors:** Dmitry Cherezov, Dmitry Goldgof, Lawrence Hall, Robert Gillies, Matthew Schabath, Henning Müller, Adrien Depeursinge

**Affiliations:** 1 Department of Computer Sciences and Engineering, University of South Florida, Tampa, Florida USA; 2 Department of Cancer Physiology, H. Lee Moffitt Cancer Center and Research Institute, Tampa, Florida USA; 3 Department of Cancer Epidemiology, H. Lee Moffitt Cancer Center and Research Institute, Tampa, Florida USA; 4 Institute of Information Systems, University of Applied Sciences Western Switzerland (HES-SO), Sierre, Switzerland; 5 University of Geneva, Geneva, Switzerland; 6 Biomedical Imaging Group, École polytechnique fédérale de Lausanne (EPFL), Lausanne, Switzerland

## Abstract

We propose an approach for characterizing structural heterogeneity of lung cancer nodules using Computed Tomography Texture Analysis (CTTA). Measures of heterogeneity were used to test the hypothesis that heterogeneity can be used as predictor of nodule malignancy and patient survival. To do this, we use the National Lung Screening Trial (NLST) dataset to determine if heterogeneity can represent differences between nodules in lung cancer and nodules in non-lung cancer patients. 253 participants are in the training set and 207 participants in the test set. To discriminate cancerous from non-cancerous nodules at the time of diagnosis, a combination of heterogeneity and radiomic features were evaluated to produce the best area under receiver operating characteristic curve (AUROC) of 0.85 and accuracy 81.64%. Second, we tested the hypothesis that heterogeneity can predict patient survival. We analyzed 40 patients diagnosed with lung adenocarcinoma (20 short-term and 20 long-term survival patients) using a leave-one-out cross validation approach for performance evaluation. A combination of heterogeneity features and radiomic features produce an AUROC of 0.9 and an accuracy of 85% to discriminate long- and short-term survivors.

## Introduction

Computed Tomography (CT) is widely used in early detection, diagnosis and treatment planning of lung cancer^1,2^. Using standard-of-care CT images, quantitative image features such as location, spiculation, size, calcification, density (intensity), necrosis and texture of a nodule can be extracted. Radiomics is the conversion of images to structured data and the resulting quantitative features can be used in mathematical models, often learned, for finding a dependence or inter-relationships between features and a medical question such as nodule malignancy, tumor aggressiveness and prediction of treatment response^3–5^. The second role of radiomics is the extraction of features that represent information that is not typically found from CT images by the human eye alone^6–9^ or that cannot easily be quantified.

One of the well-known characteristics of cancer is tumor heterogeneity. Hence, small biopsy specimens may not be representative of a whole tumor. Moreover, tumor histology often changes over time. This makes habitat detection a subtle process. Up-to-date habitat detection using radiomic methods can be divided into two categories.

Multi-parametric or multi-modality methods such as T_1_, T_2_, Flair MRI imaging^10–13^ or PET/CT imaging^14–17^ provide enough data for the detection of physiologically similar sub-regions (“habitats”) within a nodule or a tumor. Single-modality imaging provides less information. In this case radiomic texture features associated with heterogeneity of a nodule are used^18–25^.

**Figure 1:**
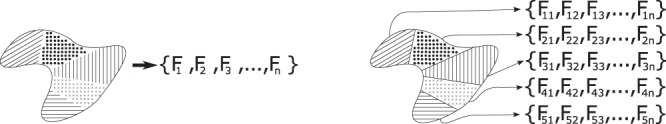
Schematic representation of a feature computation result in a workflow where a nodule is considered as a homogeneous object (on the left) and a feature computation result when heterogeneity is used for division of a nodule into habitats (on the right).

Features associated with heterogeneity of a nodule have one common characteristic: they compute texture signatures across the entire nodule (see Fig. 1). Knowing that cancer is heterogeneous and assuming that CT texture represents tissue types with different histology subtypes, we can conclude that computation of texture signatures in this case is averaging texture features of nodule regions with possibly different histology. As a result, the averaged texture features may not represent any individual habitat within a nodule correctly. In this case, the texture of each nodule is considered as a unique pattern, which makes the classification process more complicated^26^. For example, consider four habitats that have unique texture signatures: A, B, C and D. Each nodule contains two habitats: AA, AB, AC, etc. If we compute the texture signature of an AB nodule, the result will be different from the individual A and B habitat texture signatures. This difference is the result of averaging texture signatures. Thus, each unique combination of habitats in our case has a unique texture signature.

In this work, we present an approach where we compute circular harmonic wavelets for small patches within a nodule, cluster patches in order to define sub-regions of a nodule with similar patterns (habitats) and use information about the clusters and their texture signatures to describe a nodule (Fig. 1). This approach was used to classify nodules into benign and malignant. In addition, we used a dataset with 40 patients diagnosed with adenocarcinoma to evaluate how effective the approach is for classification of tumor aggressiveness.

## Materials and Methods

### Datasets

In this paper, we experimented with two datasets. First, we used the National Lung Screening Trial (NLST) dataset to evaluate how heterogeneity differentiates nodules from lung cancer patients from nodules among non-lung cancer patients. Second, we used patients from the H. Lee Moffitt Cancer Center & Research Institute diagnosed with lung adenocarcinoma for training and testing to predict patient survival time. These two datasets were chosen to allow comparisons with earlier work^3,25^ and to show that the approaches generalize to different datasets. There are labels available for the nodules and tumor images used in the study, but the pixel level ground truth is not available.

#### National Lung Screening Trial

NLST was a randomized trial of 53,439 patients that compared LDCT (Low Dose CT) vs. standard chest x-rays. After an initial screening (T0), follow-up screenings (T1 and/or T2) were conducted in intervals of approximately one-year. If at T1 a patient was diagnosed with cancer, he/she started treatment and did not have a follow-up screening. According to the screening protocol, a screen was considered positive if a non-calcified nodule (NCN) had the longest diameter (LD) larger than 4 mm. For positive screenings, radiologists provided a clinical description such as location, margin etc.

We extracted two cohorts from NLST^27^. Cancer patients in the training cohort had a positive (non-cancer) screening at time 0 and were diagnosed with cancer on the first follow-up (N = 104). Cancer patients in the test cohort had a positive, so non-cancer screening at time 0 and time 1. They were diagnosed with cancer at time 2. For each cancer patient, two non-cancer subjects were selected by demographic criteria: the same age, sex, and other available criteria. Finally, we excluded cases with technical problems or other challenges that prevented analysis of nodules. When removing a cancer patient from the dataset the corresponding non-cancer patients remained. There are 253 patients in the training cohort (83 cancer and 170 non-cancer patients) and 207 patients in the test cohort (73 cancer and 135 non-cancer patients).

Labels for the dataset represent patient diagnosis during the trial.

#### Lung Adenocarcinoma Dataset

At the H. Lee Moffitt Cancer Center & Research Institute, 276 patients with Non-Small Lung Cancer were selected. Inclusion/exclusion criteria were: (1) Diagnosed with Lung cancer; (2) Pre-surgery contrast-enhanced CT imaging performed at the H. Lee Moffitt Cancer Center & Research Institute; (3) At least 2 years of follow-up information is known; (4) Patients with all TNM stages accepted; (5) No mix of cancer types for a patient.

Out of 276 patient 86 were diagnosed with Adenocarcinoma. From the Adenocarcinoma subset, two quartiles were selected to represent distinct phenotypes: aggressive phenotype associated with short term survival patients and a non-aggressive phenotype associated with long term survival patients. It was recognized that without a time gap around the class cutoff, it was likely that significant confusion would occur near any cutoff. For short term survival group selection criteria was survival time less than 500 days. For the long term survival group selection criteria was survival time greater than 1000 days. Survival time was computed as the difference between the day of pre-surgery imaging and the last day of contact or the day of patient’s death.

Among 86 patients with adenocarcinoma 20 patients survived from 103 to 498 days. Mean survival time was 288 days. These patients were labeled short-term survivors. 20 patients survived from 1351 to 2163 days with the mean survival time of 1569 days. These patients were labeled long-term survivors.

Overall, 40 patients were used for classification of long term survivors and short term survivors. Demographic information for the patients is shown in Table 1.

**Table 1:** Demographic Summary of Patients in the Adenocarcinoma Data Set.

Characteristics	Short Survival Class	Long survival class	P Value
Age, mean (SD)	69 (8.07)	64.45 (9.75)	0.1161 (Unpaired student t-test)
Sex, N (%)			0.2049 (Fisher exact test)
Male	12 (60%)	7 (35%)	
Female	8 (40%)	13 (64%)	
Race			1 (Fisher exact test)
White	20 (100%)	20 (100%)	
Black, Asian, and Others	0 (0%)	0 (0%)	
Ethnicity, N (%)			1 (Fisher exact test)
Hispanic or Latino	1 (5%)	0 (%)	
Neither Hispanic/Latino and unknown	19 (95%)	20 (100%)	
Histology, N (%)			
Adenocarcinoma	20 (100%)	20 (100%)	
Squamous cell carcinoma	0 (100%)	0 (100%)	
Other, NOS, unknown	0 (100%)	0 (100%)	
Stage, N (%)			0.07346 (Mann-Whitney U test)
I	4 (20%)	10 (50%)	
II	5 (25%)	5 (25%)	
III	10 (50%)	3 (15%)	
IV	1 (5%)	2 (10%)	
Carcinoid, unkown	0 (0%)	0 (0%)	
Tobacco Use, N (%)			
Moderate (1–2 PPD)	4 (20%)	4 (20%)	
Light (<1PPD)	0 (0%)	1 (5%)	
HIST	12 (60%)	12 (60%)	
None	0 (0%)	3 (15%)	
Cigarettes Nos	4 (20%)	0 (0%)	

#### Pre-processing

For both datasets, segmentations of tumors were obtained at the H. Lee Moffitt Cancer Center where a qualified radiologist applied a semi-automated 3D segmentation algorithm^28^.

2D wavelet features were used in the work of this paper. Thus, for each primary nodule from both datasets, we extracted one slice where the segmentation area was the largest. We re-sampled each selected slice such that the XY spacing became equal to 0.5 mm. In the case that, a nodule segmentation area is less than a single patch area, the original slice segmentation was used as a patch and we considered that a nodule had only one habitat. For re-sampling, we used the bicubic interpolation algorithm implemented in Matlab R2016b^29^.

## Methods

**Figure 2:**
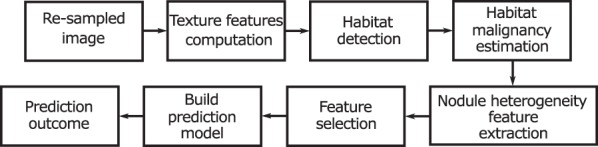
Suggested workflow for heterogeneity estimation.

In this section, we describe the proposed workflow with dedicated subsections for each step. Figure 2 shows the workflow as a diagram. First, we describe texture feature computation, patch extraction within a nodule and computation of the convolution response for a patch. Second, we explain how we defined the number of habitats for each nodule, the habitats of multiple patches and the habitat texture response. We assume that malignancy of habitats within a nodule can vary. Finally, we explain how we used texture responses of habitats to evaluate their malignancy for training and test cohorts in detail.

In order to classify patients, we extracted quantitative features that described the heterogeneity of a nodule. These features were used in the patient classification experiments. Performance of the features is shown in the Results section.

### Circular Harmonic Wavelet Features

We chose to use Circular Harmonic Wavelets (CHW) to characterize local texture properties of a tumor image *f*(*x*, *y*)^30^. CHWs quantify the amount of local circular frequencies, similar to local binary patterns (LBP)^31^. An interesting property of CHWs is their ability to characterize image directions in a rotation-invariant fashion at a very low computational price^32^. This allows quantification of benign or malignant tissue structures independently of their local orientations. CHWs of order *n* are constructed in the Fourier domain as

1 $${\hat{\varphi }}^{(n,s)}(\rho ,\,\theta )=\hat{h}{\mathrm{(2}}^{s}\rho ){{\rm{e}}}^{{\rm{j}}n\theta },$$

where (*ρ*, *θ*) denotes the polar coordinates in the Fourier domain and $\hat{h}(\rho )$ is a purely radial bandpass function controlling the wavelet analysis at scale *s*. Simoncelli’s isotropic collection of wavelets was used for $\hat{h}(\rho )$ ^33^, which proved to work well for analyzing lung tissue in CT^34^. At a given position (*x*_0_, *y*_0_), the representation obtained from the collection of the complex magnitudes of the scalar products $|\langle f,{\varphi }^{(n,i)}\rangle |$ characterizes the local circular frequencies in *f* of order *n* = −*N*:*N* at a scale *s* = 1:*S* and is locally rotation invariant^35^. This yields a collection of positive response maps having the same dimension as the domain of *f*. Features are obtained by averaging each response map over patches.

**Figure 3:**
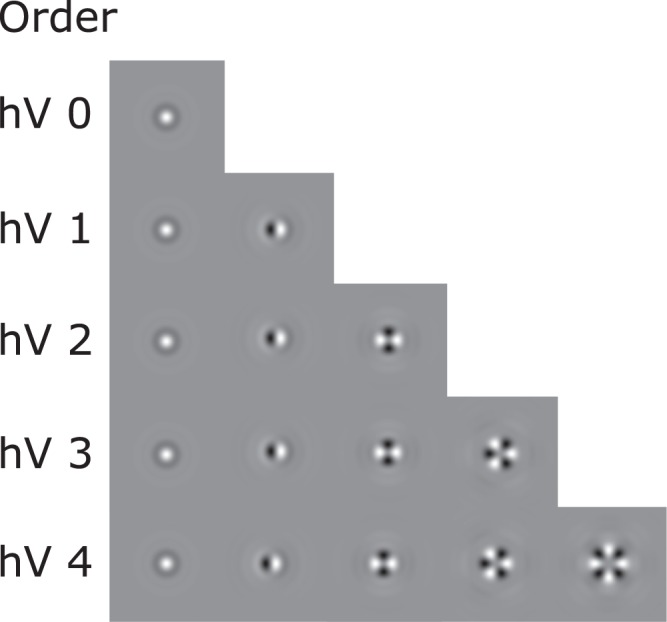
Set of Circular Harmonics filters used for texture signature computation (hV–Harmonic Vectors).

We consider five collections of circular harmonics. Figure 3 shows their impulse responses. For each frequency, in a collection, we consider three scales (*S* = 3).

For each pixel within the segmentation we get a convolution response. In order to detect texture patterns, we divide nodules into circular patches with a radius of 3 mm (6 pixels), a shift of 1.5 mm (3 pixels) and we average the absolute values of the wavelet responses within a patch. The choice of the radius of 3 mm was based on Lung-RADS (Reporting and Data System) categories. The procedure was repeated for each set of harmonic vectors (hV) individually and as a result, we obtained five sets of patches for each nodule where each patch has a different number of texture features. The number of texture features extracted from each collection shown in Fig. 3 is equal to 3, 9, 15, 21 and 27 respectively.

### Habitat Detection

After computing a set of wavelet features (for each set of harmonic vectors) within each patch the k-means++ algorithm^36,37^ was applied to identify regions with a similar texture. The number of texture features for each set is provided above. The number of clusters was estimated with the gap criterion clustering evaluation method^38^. The maximum number of possible clusters is limited to 15. As a result of the habitat detection step, we obtained five sets of habitats for each nodule with respect to five sets of harmonic vectors.

### Habitat Malignancy Estimation

After habitat detection, each nodule is represented as a set of texture signatures for habitats (Fig. 1). In this work we assume that a difference in habitat histology can be described with texture patterns. To estimate the probability that a particular habitat belongs to a given class, we applied a leave-one-out cross-validation (LOOCV) on the training cohort.

**Figure 4:**
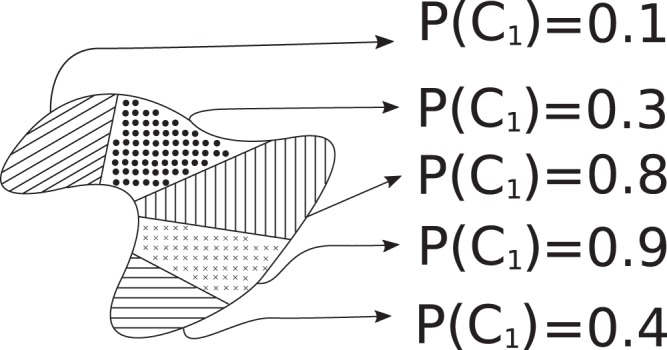
Example of the malignancy/aggressiveness probability assignment for habitats in a nodule.

We excluded from the training cohort one patient. Texture signatures from all the other patients with the corresponding labels were used for training. After training, the excluded patient’s texture signatures were used for testing. The classifier produced a pseudo-probability for each signature (Fig. 4). The procedure was repeated for each patient in the training cohort. We collected these pseudo probabilities to describe a nodule and see if this information can be used for the computation of quantitative features and a nodule level classification task.

Several studies showed that Random Forests outperform other classifiers in Radiomics experiments^3,39,40^. Thus we chose random forests for classification, where the fraction of decision trees that voted that a habitat is malignant/aggressive to the total number of decision trees is recorded. This fraction is considered as a pseudo-probability of malignancy/aggressiveness.

To evaluate malignancy/aggressiveness of habitats in the test cohorts we used all signatures from the training cohort for training with the assigned labels of the corresponding patient. After this step, for each habitat in the training and the test cohorts, we estimate the probability of it being malignant or aggressive. Habitat malignancy/aggressiveness estimation for the training and test cohorts are repeated for each set of habitats.

### Nodule Heterogeneity Feature Extraction

The detection of habitats within a nodule provides much information about its heterogeneity. Nevertheless, it makes it impossible to compare the texture of each nodule directly because the number of habitats differs and because we do not know the relationship between the habitat area, the level of malignancy/aggressiveness of a habitat and the malignancy of the nodule itself.

We produced 15 quantitative radiomics features. These features are statistical information of habitat area, habitat pseudo probability of malignancy/aggressiveness and variety in habitat texture signatures of the nodule. Table 2 shows the names of the features and the corresponding description. Heterogeneity features were extracted from all patients and were used for nodule classification.

**Table 2:** Heterogeneity features description.

Feature name	Feature description
min P	Minimum value of the malignancy pseudo probability of a habitat in a nodule.
max P	Maximum value of the malignancy pseudo probability of a habitat in a nodule.
mean P	Mean value of the malignancy pseudo probability of a habitat in a nodule.
median P	Median value of the malignancy pseudo probability of a habitat in a nodule.
min A ratio	Minimum value of a habitat area in a nodule.
max A ratio	Maximum value of a habitat area in a nodule.
mean A ratio	Mean value of a habitat area in a nodule.
median A ratio	Median value of a habitat area in a nodule.
min disjoint A ratio	Minimum value of a habitat area in a nodule in the case of disjoint parts of a habitats being considered as different habitats.
max disjoint A ratio	Maximum value of a habitat area in a nodule in the case of disjoint parts of a habitats being considered as different habitats.
mean disjoint A ratio	Mean value of a habitat area in a nodule in the case of disjoint parts of a habitats being considered as different habitats.
median disjoint A ratio	Median value of a habitat area in a nodule in the case of disjoint parts of a habitats being considered as different habitats.
number of clusters	Total number of habitats in a nodule.
mean centroids dist	Computing mean value of habitat texture signatures for a nodule–nodule texture signature. The result is mean Euclidean distance from the nodule texture signature to its habitats texture signatures.
dist std centroids	Standard deviation of habitat texture signatures.

## Results

As a baseline for all experiments we use the results of the classification with 3D radiomics features extracted using the Definiens application and consisting of size, location, intensity and texture features (219 features) computed on the entire 3D ROI^41^. Balagurunathan *et al*. described all categories and features in detail in their work^42^. During feature computation, CTs were not resampled to have uniform spacing.

Heterogeneity features are 2D features and only texture information was used to define them. We combined 2D heterogeneity features and 3D radiomics features to test if such a fusion improves classification performance on the NLST dataset.

For experiments with only the heterogeneity features or their combination with 3D Definiens features, we tested heterogeneity features computed by each frequency collection individually (Fig. 3).

To reduce the number of features we applied the ReliefF feature selector^43–45^ to select the best 5, 10 features and the minimum redundancy maximum relevance feature selector^46^. Experiments with no feature selection were used as a baseline. With the exception of Naive Bayes, all classifiers used do implicit feature selection. Reducing the number of features with a feature selector resulted in better performance. As a subset of the Definiens features we consider features that were shown to be stable on the RIDER dataset and separately features stable on the training cohort^41^. As the set of classifiers, we selected Naïve Bayes^47^, J48^48^, JRIP^49^, Random Forests^50^ and SVMs with a linear and a radial basis function kernel^51^. All the experiments were executed in Weka, version 3.8.1.

### Lung Cancer vs. Non-Lung Cancer in NLST

Three experiments were based on the NLST dataset. First, we used patient screening results at the time of diagnosis: the training cohort at time 1 vs. test cohort at time 2. Second, we used patient CT screening one year ahead of diagnosis to evaluate the heterogeneity of malignant nodules before they were marked as cancer: the training cohort at time 0 vs. test cohort at time 1. Finally, we used the training cohort at time 0 vs. test cohort at time 0, which means that for training we used CT screenings of nodules a year ahead of diagnosis and for testing we use CT screenings of nodules two years ahead of diagnosis. The AUROC of classifiers was considered as the primary performance measure. Heterogeneity helped when combined with 3-D features in all cases except predicting two years in advance. Table 3 shows feature sets, feature selectors and classifiers producing the best AUROCs. The NLST combined model AUROC was compared to the Definiens model performance in R by using the pROC library^52^ and the comparison algorithm of DeLong *et al*.^53^. The P-value of AUROC differences is 0.2215.

**Table 3:** Overview of classification models that produce the best AUROC.

Screening time	Feature type	Feature set	Feature Selector	Classifier	AUROC	Accuracy (%)
Training Set Diagnosis Test Set Diagnosis	Heterogeneity	*hV*_4_	RfF 10	RFs	0.77	72.95
	Definiens	All 219 features	mRMR 17*	RFs	0.83	78.77
	Combined	Training st. +*hV*_4_	none	RFs	0.85	81.64
Training set Diagnosis - 1 year Training set Diagnosis - 1 year	Heterogeneity	*hV*_3_	none	*SVM*_*lin*_	0.69	74.88
	Definiens	RIDER st.	RfF 10	RFs	0.79	75
	Combined	All 219 +*hV*_2_	mRMR 25*	RFs	0.79	74.4
Training set Diagnosis - 1 year Training set Diagnosis - 2 years	Heterogeneity	*h*_*V*1_	RfF 10	RFs	0.67	65.7
	Definiens	RIDER st.	RfF 5	RFs	0.78	74.06
	Combined	RIDER st. +*h*_*V*0_	RfF 10	RFs	0.78	70.53

The first column defines the time point of the CT screening that was used in the training and test cohorts. The second column defines which feature set was extracted for a given CT screening. Heterogeneity refers to only 15 texture heterogeneity features, Definiens refers to 219 features extracted in Definiens. Combined refers to the fusion of Definiens and heterogeneity features. The feature subset column defines the order of the Circular Harmonic vectors that were used to extract texture features or a subset of the Definiens features claimed to be stable on the RIDER or training datasets. The feature selector column defines one of the feature selectors that produces the best performance. There can be no feature selector, ReliefF (RfF) with top 10 or 5 ranked features or the minimum redundancy maximum relevance (mRMR) feature selector. The classifier column defines, which of the tested classifiers performed the best. From the table we can see that most of the time random forests (RFs) outperformed other classifiers. Finally, the last two columns refer to AUROC and accuracy of the corresponding model. ^*^Weka v.3.8.1 provides mRMR algorithm whose implementation defines the optimal number of features for a particular dataset in terms of redundancy and relevance. As a result, the selected number of features varies.

### Survival time prediction in the Adenocarcinoma dataset

Patient stage information from Table 1 was used to evaluate baseline performance using clinical data due to the significant difference in patient survival time. If a patient stage is used as a quantitative feature to differentiate long/short term survival the AUROC is equal to 0.67 (Supplementary Fig. S1). If stages I/II are considered as early stages and stages III/IV are considered as late stages, then the split produces a confusion matrix which leads to an accuracy of 65% (Supplementary Table S1).

Due to the small number of patients in the adenocarcinoma dataset, we applied cross-validation for the performance evaluation. Because habitat aggressiveness estimation was performed with leave-one-patient-out, the same concept was applied when testing nodules.

Cross-validation for this dataset with the Definiens features showed an AUROC = 0.71 and an accuracy = 77.5%^25^. Using heterogeneity features alone provides the best AUROC of 0.80 with the corresponding accuracy of 85%. The enhanced contrast likely enabled better habitat definition. Again, the combination of features provided the best results.

The combined model with the best performance from the Lung Adenocarcinoma Dataset is based on a subset of Definiens features that are shown to be stable and reproducible by test-retest analysis on the RIDER dataset^41^. There are 23 features in the RIDER subset of features. In addition to the 23 RIDER features, there are 15 heterogeneity features. Out of 38 total features, the ReliefF algorithm selected the top 5 predictive features. Random Forests for a classification task with N features used the square root of N features for building a decision tree. This means that a random set of sqrt(N) features are chosen with the best of them selected for the test at an internal node. This is an implicit form of feature selection.

Table 4 shows the classification models that perform best for particular feature sets with the corresponding AUROC and accuracy. The Adenocarcinoma Combined model AUROC is compared to Definiens model performance in R by using the pROC library^52^ and the DeLong *et al*.^53^ comparison algorithm. The P-value of AUROC differences was 0.04924.

**Table 4:** Comparison of Adenocarcinoma aggressiveness estimation results using the heterogeneity and Definiens features.

Feature type	Feature set	Feature selector	Classifier	AUROC	Acc. (%)
Staging	NA	NA	NA	0.67	65
Heterogeneity	*hV*_3_	mRMR 1*	J48	0.80	85
Definiens	all 219 features	RfF 5	J48	0.71	77.5
Combined	RIDER +*hV*_3_	RfF 5	RFs	0.90	85

^*^Weka v.3.8.1 provides the mRMR algorithm, which defines the optimal number of features for a particular dataset in terms of redundancy and relevance. As a result, the selected number of features varies.

## Discussion

The hypothesis of this work was that CT screening data can be used not only for the description of a nodule but it is a source of information to define habitats within nodules and that the level of heterogeneity is able to help identify malignancy and aggressiveness of tumors.

As can be seen from Table 3, the results of the three experiments performed on NLST for all features highlight that the highest performance was achieved when using images at the time of diagnosis (training set at T1 vs. test set at T2). The next best performance was reached where both cohorts used data one year ahead of diagnosis (training set at T0 vs. test set at T1). Finally, the worst result was obtained when we used as for training the CT screenings of nodules one year ahead of diagnosis and for testing the CT screenings of nodules that were taken two years ahead of diagnosis. Clinically, the complexity of these questions is on the same order, so from the simplest to the most complicated.

Heterogeneity helps performance most at the time of diagnosis. In addition, technically, it is hard to evaluate heterogeneity of small nodules due to the limited spatial resolution of CT volumes. Most of the nodules at the time of the initial CT screening have a longest diameter of less than 15 mm. In this work, we used patches with a radius of 6 mm. Thus, with very few patches we cover entire nodules and thus we may miss all the information about local habitats. At the time of diagnosis, nodules have grown and thus the performance of habitat detection increases.

In this regard, the Adenocarcinoma dataset is a better choice, as the nodules are larger and contrast is stronger. Thus, the proposed method can better leverage habitat heterogeneity information. Longest diameter mean and standard deviation values for the NLST dataset were 11.06 mm and 7.62 mm respectively. For Adenocarcinoma these parameters were 34.09 mm and 17.4 mm respectively. In addition, CT imaging of Adenocarcinoma dataset patients was performed with use of a contrast agent, which highlights CT texture inside a tumor.

As we can see from Table 4, in the experiment where we used only heterogeneity features the mRMR feature selector selected only one feature. The feature name is “Min P” which is explained in Table 2. After splitting nodules into habitats and computing pseudo-probability of aggressiveness for habitats with Random Forests we selected the minimum value. Just by using this value for nodule aggressiveness classification we got an AUROC of 0.8 and an Accuracy of 85%. This may be the result of the fact that we computed texture signatures for habitats individually.

## Conclusions

In this paper, we propose a method for revealing tumor habitats from texture heterogeneity. We use this heterogeneity to classify lung cancer malignancy and aggressiveness. We analyze classification abilities of heterogeneity on two datasets and compared it with 3D features from Definiens based on the entire tumor volume (i.e., not considering tumor habitats). First, we applied heterogeneity for classifying cancer and non-cancer patients in the NLST screening dataset. The best results were obtained when using CT images at the time of diagnosis. When using Definiens features only (219 features), the best AUROC is 0.83. When using the proposed 15 2D heterogeneity features, the best AUROC is 0.79. Combining the two sets of features achieved the top AUROC of 0.85. This small gain suggests that NLST nodules are relatively small to fully benefit from the proposed heterogeneity analysis but it does add important information. To this end, we evaluated heterogeneity in adenocarcinoma patient survival time prediction, where nodules are much larger. In this dataset, the best AUROC was obtained when the model was based on the heterogeneity features (AUROC = 0.8), whereas the global Definiens features were mixing distinct habitats and only achieved the highest AUROC of 0.71. Combining heterogeneity features and the Rider subset of features resulted in a statistically significant improved AUROC of 0.9.

## Supplementary information

Revealing Tumor Habitats from Texture Heterogeneity Analysis for Classification of Lung Cancer Malignancy and Aggressiveness Supplementary info

## Data Availability

National Lung Screening Trial dataset^54^ and Adenocarcinoma dataset^55,56^ are available at The Cancer Imaging Archive^57^. The Matlab code for heterogeneity detection described in the section on Circular Harmonic Wavelet Features and the section on Habitat Detection is available on the GitHub server^58^.
