# Supplementary material for: Revealing Tumor Habitats from Texture Heterogeneity Analysis for Classification of Lung Cancer Malignancy and Aggressiveness
Source: Sci Rep. 2019 Mar 14;9:4500. doi: 10.1038/s41598-019-38831-0 (PMC6418269; doi:10.1038/s41598-019-38831-0)
Supplement: Supplementary file 1 — Revealing Tumor Habitats from Texture Heterogeneity Analysis for Classification of Lung Cancer Malignancy and Aggressiveness Supplementary info [file 41598_2019_38831_MOESM1_ESM.docx]

**Supplementary Information Article in *Scientific Reports***

# Revealing Tumor Habitats from Texture Heterogeneity Analysis for Classification of Lung Cancer Malignancy and Aggressiveness

# Dmitry Cherezov, Dmitry Goldgof, Lawrence Hall, Robert Gillies, Matthew Schabath, Henning Mu¨ ller, and Adrien Depeursinge

# Supplementary Discussion S1

# Adenocarcinoma dataset stage analysis

If stage information is used as a feature for survival then AUROC is equal to 0.67 (Supplementary Fig. S1). If stages I/II are considered as early stages and stages III/IV are considered as late stages, then the split produces a confusion matrix (Supplementary Table S1) which leads to an accuracy of 65%.

**
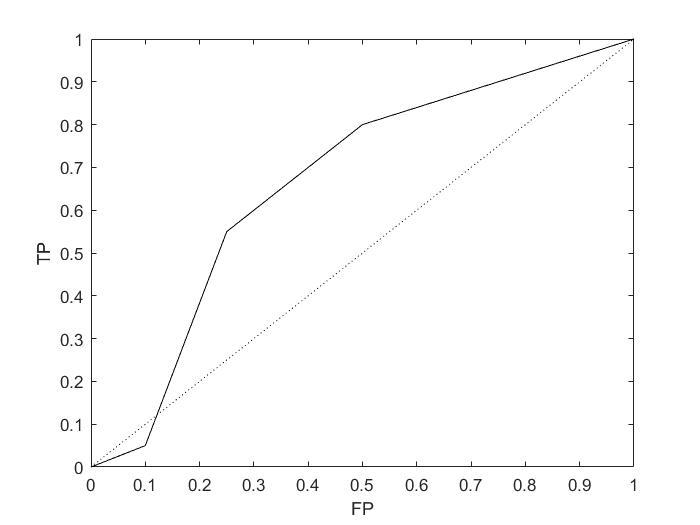
**

**Supplementary Figure S1**. **Receiver Operator Curve for stage feature.**

|  | early stages | late stages |
| --- | --- | --- |
| LTS | 15 | 5 |
| STS | 9 | 11 |

**Supplementary Table S1**. **Confusion matrix for long/short term survival from early and late stages.** LTS – Long Term Survivals. STS – Short Term Survivals.

# Supplementary Discussion S2

# National Lung Screening Trial patient list

# Cohort 1

# 100012, 100147, 100186, 100913, 100954, 100965, 101012, 101068, 101444, 101467, 101859, 101996, 102038, 102082, 102140, 102315, 102371, 102488, 102516, 102607, 102620, 102629, 102658, 103303, 103361, 103458, 103721, 104208, 104250, 104302, 104386, 104683, 104705, 104815, 105042, 105071, 105085, 105148, 105205, 105340, 105526, 105808, 105941, 105949, 105974, 106058, 106990, 107058, 107232, 107237, 107434, 107682, 107955, 108352, 108392, 108474, 108504, 108527, 108577, 108714, 108834, 108937, 109237, 109345, 109538, 109573, 109589, 109878, 109897, 109957, 110253, 110522, 110846, 110878, 110987, 111121, 111200, 111702, 112183, 112258, 112390, 112575, 112901, 112957, 113308, 113665, 113857, 114263, 114323, 114958, 115017, 115174, 116279, 116289, 116329, 116332, 116420, 116837, 117025, 117453, 117820, 117947, 118012, 118145, 118226, 118681, 118743, 119173, 119343, 119358, 119743, 119924, 119958, 120358, 120378, 120556, 120762, 120790, 120954, 121130, 121169, 121438, 121738, 121967, 121999, 122352, 122364, 122392, 122492, 122541, 122549, 122652, 122836, 122965, 123018, 123344, 123459, 123515, 123559, 123740, 123909, 124436, 124864, 125378, 125923, 125982, 126144, 126254, 126265, 126792, 126955, 127000, 127400, 127414, 127619, 127886, 128024, 128275, 128564, 128829, 129140, 129553, 129741, 130033, 130139, 130231, 130352, 130544, 130689, 130692, 130869, 131122, 131124, 131174, 131465, 131486, 131537, 131800, 131979, 132526, 132529, 132969, 133700, 133786, 134120, 134257, 200056, 200397, 200525, 201632, 201701, 201979, 202570, 203168, 203231, 203372, 203512, 203578, 203852, 203981, 204335, 204438, 204604, 204694, 204711, 204836, 204859, 205415, 205617, 205687, 205806, 205900, 206344, 206359, 206483, 206809, 207584, 207647, 207782, 207857, 208287, 208794, 208801, 209029, 209095, 209676, 209831, 210231, 210528, 211423, 211696, 212222, 212827, 212831, 212849, 213038, 213413, 213544, 213630, 213754, 213769, 214097, 214377, 214487, 214632, 214728, 215151, 216160, 216308, 216940, 217245, 217877, 218248, 218391, 218666

# Cohort 2

# 100005, 100095, 100414, 100629, 100658, 100681, 100727, 101192, 101321, 101428, 101563, 101692, 102154, 102641, 103239, 103359, 103621, 103874, 104355, 104377, 104769, 104792, 104871, 104999, 105617, 105632, 106194, 106226, 106553, 106957, 107211, 107579, 107910, 108061, 108320, 108461, 108539, 108600, 108921, 109031, 109127, 109965, 110802, 110919, 110994, 111452, 111454, 112180, 112220, 112506, 112606, 112786, 112961, 113014, 113820, 114517, 114656, 114796, 115020, 115175, 115571, 115772, 115794, 116383, 117406, 117490, 117610, 117950, 118243, 118297, 118553, 118602, 118719, 118745, 119485, 119533, 119568, 119894, 119934, 120070, 120573, 120593, 120885, 121657, 121852, 122078, 122117, 122159, 122376, 122378, 122577, 122590, 123062, 123810, 123884, 123891, 124323, 124607, 124913, 125028, 125413, 125697, 125727, 125898, 126101, 126581, 126622, 126718, 126823, 126928, 127048, 127731, 127996, 128033, 128535, 128601, 128852, 128899, 129511, 129534, 129703, 129734, 130117, 130173, 130896, 130950, 131611, 131963, 131986, 132313, 132535, 132823, 132885, 133076, 133308, 133991, 134309, 134491, 134503, 200129, 200221, 200268, 200628, 200834, 200925, 201368, 201446, 201737, 201890, 202611, 202709, 202748, 202822, 202873, 203344, 203536, 203759, 203921, 203930, 204238, 204377, 204494, 205023, 205964, 206737, 206870, 206925, 207830, 208147, 209119, 209137, 209318, 209445, 209852, 210090, 210198, 210419, 210483, 210653, 210700, 210754, 211092, 211965, 212200, 212202, 212522, 212718, 213139, 213215, 213439, 213442, 213734, 214270, 214553, 214700, 215325, 215446, 215687, 216089, 216411, 216422, 216666, 217021, 217203, 217676, 218217, 218320, 218383, 218510, 218662
